# Successful Management of Unilateral Post-traumatic Neuropathic Mastalgia: A Case Report

**DOI:** 10.5152/TJAR.2023.21216

**Published:** 2023-02-01

**Authors:** Sukanya Mitra, Jasveer Singh, Kompal Jain, Uma Rathi

**Affiliations:** Department of Anaesthesia and Intensive Care, Government Medical College & Hospital, Chandigarh, India

**Keywords:** Neuropathic breast pain,, pain,, post-traumatic,, pulsed radiofrequency,, regional anaesthesia,, stellate ganglion block

## Abstract

Stellate ganglion block has been described in the management of postmastectomy neuropathic pain. However, its role in the treatment of posttraumatic neuropathic breast pain has not been reported in the literature. We present a case of a 40-year-old female with a chief complaint of severe debilitating pain in her right breast following trauma, refractory to oral medications including conventional analgesics, amitriptyline, pregabalin, and duloxetine. She was successfully managed after administration of ultrasound-guided stellate ganglion block and pulsed radiofrequency ablation of the stellate ganglion. It resulted in significant and prolonged pain relief leading to improved quality of life.

**Main Points**

Stellate ganglion block (SGB) had promising results in severe, refractory, intractable post traumatic neuropathic mastalgia.
Pulsed radio frequency ablation (PRF) of stellate ganglion (SG) can be used in long term management of post-traumatic neuropathic mastalgia.
Both SGB and PRF of SG resulted in better quality of life.

## Introduction

Post-traumatic neuropathic mastalgia is a rare entity. No data are present in the literature about the management of post-traumatic neuropathic mastalgia refractory to medications. Stellate ganglion block has been described for the treatment of postmastectomy neuropathic mastalgia and lymphedema in patients with breast cancer.^1-3^ However, its role in the treatment of refractory post-traumatic neuropathic mastalgia has not been described yet. Therefore, we report a case of successful management of refractory post-traumatic breast pain using ultrasound-guided stellate ganglion block and pulsed radiofrequency (PRF) ablation of stellate ganglion.

## Case Presentation

A 40-year-old female with no comorbidity presented to the surgical outpatient department with severe pain in her right breast following blunt trauma 3 months back. On clinical examination, no lump associated with any change in the skin was noticed. Ultrasound of the breast and mammogram were normal; hence, malignancy of the breast was ruled out. She was treated for fibroadenosis of the breast and was prescribed tablet diclofenac, followed by tramadol–paracetamol combination. However, the pain was refractory to these conventional analgesics. She requested an elective mastectomy due to severe and persistent pain.

The patient was referred to the pain clinic. Her pain score using a numerical rating scale was 9/10. The pain was localised to her right breast and was described as prickling, non-radiating, and aggravating at night. It was associated with a burning sensation and intermittent exacerbations, interfering with her sleep and appetite. Her breast was sensitive to light touch and clothing. On examination, allodynia was presently associated with no change in colour or temperature. She scored 6/10 on the neuropathic pain diagnostic questionnaire (DN4 questionnaire) confirming the diagnosis of neuropathic pain.^4^

She was started on tablet amitriptyline 10 mg at bedtime and tablet pregabalin 75 mg twice daily. Her NRS pain score came down to 5/10 after 2 weeks. Oral amitriptyline was increased to 25 mg and pregabalin was increased to 150 mg at bedtime and 75 mg in the morning. Her pain improved at the end of 1 month with a pain score of 3/10; however, she complained of dizziness and daytime sedation. She, being a treatment defaulter again returned to the pain clinic with her previous complaints after 2 months. She was then started on duloxetine 60 mg twice a day along with amitriptyline; however, the results were not promising.

**Figure 1.**
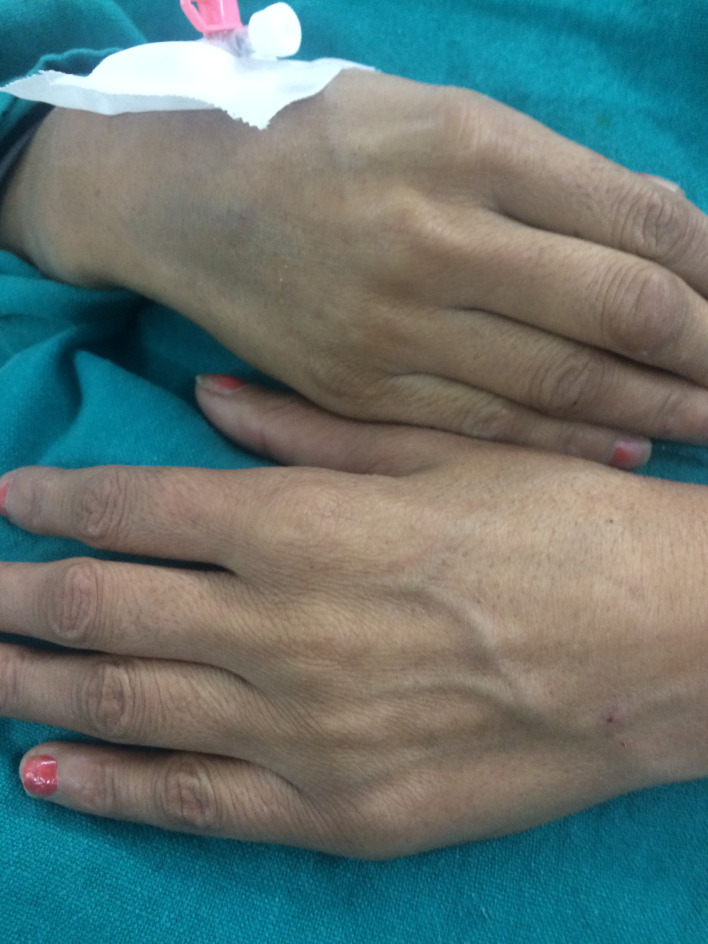
Figure demonstrating the dilated veins of right hand after successful stellate ganglion block.

After informed consent, ultrasound-guided stellate ganglion block (SGB; right), using an in-plane technique was administered with 4 mL of 0.25% bupivacaine and 40 mg of triamcinolone. Under ultrasound guidance, the tip of the needle was positioned on the longus colli muscle after penetrating the prevertebral fascia, at the level of C7. Following a negative aspiration test for blood or cerebrospinal fluid (CSF), the local anaesthetic was injected and visualised spreading in real time, dissecting the carotid artery and longus colli muscle. She developed transient ptosis (right) followed by dilatation of the veins of the right hand (Figure 1). Her NRS pain score decreased to 2/10 with improvement in her daily functioning and quality of life. She was further maintained on pregabalin 75 mg twice daily, along with amitriptyline 25 mg at bedtime.

After 2 weeks, the patient experienced pain with an NRS of 5/10. Ultrasound-guided in-plane PRF ablation of SG (right) was performed at the C7 level. It was evident by the disappearance of the anterior tubercle of the transverse process. After skin infiltration, a 5-cm long, 22-gauge radiofrequency (RF) needle, with a 5-mm active tip was introduced in-plane in lateral–medial direction carefully under ultrasound guidance to avoid penetration of the pulsating vertebral artery. The needle tip was placed under the prevertebral fascia, on the longus colli muscle. Then, a PRF was administered for 480 seconds at 42°C. After PRF and negative blood or CSF aspiration, 4 mL of 0.25% bupivacaine plus 40 mg of triamcinolone was injected through the needle before termination of the procedure. After 1 week, her pain score came down to 2/10 and lasted for a month. Over a period of the next 4 months, she received 4 such PRF treatments, along with local anaesthetic and steroid injections. At the end of 6 months, her medications were discontinued. We followed the patient for 1 year telephonically. The patient had an improved and better quality of life with no complaints of pain.

## Discussion

Stellate ganglion block has been described as a prognostic, diagnostic, or therapeutic intervention of sympathetically medicated pain involving the abnormal connection between sensory and sympathetic nervous system in regions of the head, neck and upper extremities.^1,5,6^ In addition to the sympathetic blockade, it also reduces the release of substance P in the spinal cord and plasma catecholamine in response to noxious stimuli further alleviating the nociceptive response.^7^

It has also been successfully used in the treatment of postmastectomy pain syndrome and lymphedema.^1-3^ Different imaging tools for SGB include fluoroscopy, ultrasound, magnetic resonance imaging, and computed tomography.^5^ Park et al^2^ concluded that the addition of corticosteroids in SGB may lead to faster and better pain relief by enhancing immune modulation. Similarly, in our case, significant improvement in pain and allodynia was noted after SGB using local anaesthetic and corticosteroid in intractable post-traumatic neuropathic mastalgia refractory to antiepileptics, tricyclic antidepressants, and serotonin–norepinephrine reuptake inhibitors. Following SGB, for prolonged and long-term effects, PRF ablation of SG was performed under ultrasound guidance. Similarly, Abdel Ghaffar et al^8^ concluded that PRF of second, third, and fourth thoracic dorsal root ganglion along with tamoxifen improved the quality of life in non-cyclic mastalgia.

Pulsed radiofrequency carries the advantage of little thermal injury and avoids the complications of neuritis and deafferentation pain as compared to conventional RF. Pulsed radiofrequency acts by neuromodulation and also reduces the release of substance P in response to noxious stimuli.^6,9^ In contrast, Abbas and Reyad^6^ concluded that thermal RF is more effective than pulsed RF of the stellate ganglion in postmastectomy pain syndrome. However, there was no significant difference in the quality of life or patient functional capacity. They performed RF therapy of SG under both fluoroscopic and ultrasound guidance.^6^

We used ultrasound as compared to other imaging modalities for SGB and PRF ablation of SG as it clearly visualises vascular and soft tissue structures including the thyroid gland, thyroid vessels, vertebral artery, longus colli muscle, and oesophagus, respectively, further decreasing the radiation exposure. Precise positioning of the RF needle tip and prevention of injection within the longus colli muscle under ultrasound guidance increases the success rate of PRF ablation of SG and SGB, respectively.^5^

Complications of SGB include puncture of oesophagus, trachea, vascular or neural structures, pneumothorax, thyroid injury, transient Horner’s syndrome, intravascular injection, and neuraxial, phrenic nerve or brachial plexus spread of local anaesthetic.^5^ In our case, no complication occurred although we performed the block at the C7 level as compared to the safer C6 level. Thermographic assessments revealed that SGB-C6 though safer leads to incomplete sympathetic effects as compared to the C7 level, and hence, SGB-C7 is more successful than SGB-C6.^10^

## Conclusion

The ultrasound-guided SGB is a useful treatment modality for severe, refractory, intractable post-traumatic neuropathic mastalgia. Pulsed radiofrequency ablation of the stellate ganglion can be a potential alternative for the long-term treatment of intractable post-traumatic neuropathic mastalgia, refractory to oral medications.
